# MRI versus Mammography plus Ultrasound in Women at Intermediate Breast Cancer Risk: Study Design and Protocol of the MRIB Multicenter, Randomized, Controlled Trial

**DOI:** 10.3390/diagnostics11091635

**Published:** 2021-09-08

**Authors:** Luigina Ada Bonelli, Massimo Calabrese, Paolo Belli, Stefano Corcione, Claudio Losio, Stefania Montemezzi, Federica Pediconi, Antonella Petrillo, Chiara Zuiani, Lucia Camera, Luca Alessandro Carbonaro, Andrea Cozzi, Daniele De Falco Alfano, Licia Gristina, Marta Panzeri, Ilaria Poirè, Simone Schiaffino, Simona Tosto, Giovanna Trecate, Rubina Manuela Trimboli, Francesca Valdora, Sara Viganò, Francesco Sardanelli

**Affiliations:** 1 Unit of Clinical Epidemiology, IRCCS Ospedale Policlinico San Martino, 16132 Genova, Italy; ilaria.poire@hsanmartino.it; 2 Unit of Diagnostic Senology, IRCCS Ospedale Policlinico San Martino, 16132 Genova, Italy; massimo.calabrese@hsanmartino.it (M.C.); licia.gristina@sanmartino.it (L.G.); simona.tosto@hsanmartino.it (S.T.); francesca.valdora@hsanmartino.it (F.V.); 3 Department of Radiological, Radiotherapic and Hematological Sciences, Fondazione Policlinico Universitario Agostino Gemelli IRCCS, Università Cattolica del Sacro Cuore, 00168 Roma, Italy; paolobelli06@gmail.com; 4 Breast Imaging Unit, Arcispedale Sant’Anna, Azienda Ospedaliero-Universitaria di Ferrara, 44124 Cona, Italy; stefano.corcione@unife.it (S.C.); defalco.md@gmail.com (D.D.F.A.); 5 Unit of Senology, IRCCS Ospedale San Raffaele, 20132 Milano, Italy; losio.claudio@hsr.it (C.L.); panzeri.marta@hsr.it (M.P.); 6 Unit of Radiology BT, Azienda Ospedaliera Universitaria Integrata, 37126 Verona, Italy; stefania.montemezzi@aovr.veneto.it (S.M.); lucia.camera@aovr.veneto.it (L.C.); 7 Department of Radiological, Oncological and Pathological Sciences, Università degli Studi “La Sapienza”, 00161 Roma, Italy; federica.pediconi@uniroma1.it; 8 Radiology Unit, Istituto Nazionale dei Tumori IRCCS Fondazione G. Pascale, 80131 Napoli, Italy; a.petrillo@istitutotumori.na.it; 9 Institute of Radiology, Azienda Ospedaliera Universitaria “Santa Maria della Misericordia”, Università degli Studi di Udine, 33100 Udine, Italy; chiara.zuiani@uniud.it; 10 Unit of Radiology, IRCCS Policlinico San Donato, 20097 San Donato Milanese, Italy; luca.carbonaro@unimi.it (L.A.C.); simone.schiaffino@grupposandonato.it (S.S.); francesco.sardanelli@unimi.it (F.S.); 11 Department of Radiology, Grande Ospedale Metropolitano Niguarda, 20162 Milano, Italy; 12 Department of Oncology and Hemato-Oncology, Università degli Studi di Milano, 20122 Milano, Italy; 13 Department of Biomedical Sciences for Health, Università degli Studi di Milano, 20133 Milano, Italy; andrea.cozzi1@unimi.it (A.C.); trimboli.rm@gmail.com (R.M.T.); 14 Mammography Center, Radiology Unit, Policlinico Sant’Orsola–Malpighi, 40138 Bologna, Italy; 15 Department of Diagnostic Imaging, Fondazione IRCCS Istituto Nazionale dei Tumori, 20133 Milano, Italy; giovanna.trecate@istitutotumori.mi.it (G.T.); sar.vigano@gmail.com (S.V.); 16 Breast Imaging and Screening Unit, Department of Radiology, Humanitas Clinical and Research Center—IRCCS, 20089 Rozzano, Italy

**Keywords:** intermediate breast cancer risk, screening, contrast-enhanced magnetic resonance imaging, mammography, ultrasound

## Abstract

In women at high/intermediate lifetime risk of breast cancer (BC-LTR), contrast-enhanced magnetic resonance imaging (MRI) added to mammography ± ultrasound (MX ± US) increases sensitivity but decreases specificity. Screening with MRI alone is an alternative and potentially more cost-effective strategy. Here, we describe the study protocol and the characteristics of enrolled patients for MRIB feasibility, multicenter, randomized, controlled trial, which aims to compare MRI alone versus MX+US in women at intermediate breast cancer risk (aged 40–59, with a 15–30% BC-LTR and/or extremely dense breasts). Two screening rounds per woman were planned in ten centers experienced in MRI screening, the primary endpoint being the rate of cancers detected in the 2 arms after 5 years of follow-up. From July 2013 to November 2015, 1254 women (mean age 47 years) were enrolled: 624 were assigned to MX+US and 630 to MRI. Most of them were aged below 50 (72%) and premenopausal (45%), and 52% used oral contraceptives. Among postmenopausal women, 15% had used hormone replacement therapy. Breast and/or ovarian cancer in mothers and/or sisters were reported by 37% of enrolled women, 79% had extremely dense breasts, and 41% had a 15–30% BC-LTR. The distribution of the major determinants of breast cancer risk profiles (breast density and family history of breast and ovarian cancer) of enrolled women varied across centers.

## 1. Introduction

Mammography (MX) represents the primary screening tool for breast cancer, but its preventive impact is not fully satisfactory. Considering the screening age range, MX yields an estimated breast cancer mortality reduction of about 30% in the target population, with a beneficial effect persisting for at least 10 years [1,2]. However, even for women who regularly adhere to a screening program, risk reduction brought about by MX screening remains approximately 40% [3]. Such limited efficacy has been attributed to both the intrinsic limitations of MX and the highly variable biological characteristics of breast cancer [4], as well as to women’s individual characteristics such as age and breast density (BD). A high BD is an independent breast cancer risk factor for both premenopausal and postmenopausal women [5,6], and it also reduces MX sensitivity (masking effect), resulting in an increased interval cancer rate [7,8,9]. While tomosynthesis, contrast-enhanced magnetic resonance imaging (MRI), and contrast-enhanced mammography may all display increased sensitivity, compared to MX, especially for small breast cancers [10,11,12], a more sensitive test could detect more small tumors only because they are growing slowly, potentially leading to an increase in overdiagnosis [13].

In high-risk women, the addition of MX to MRI did not substantially increase sensitivity [14,15,16,17,18]. Moreover, supplementary screening breast ultrasound (US) or MRI in addition to MX in these women resulted in a higher cancer detection rate [16,19] but also increased the false-positive rate [16,20]. In this context, any attempt to improve screening efficacy by increasing the sensitivity of the process/test(s) needs to be carefully evaluated, since it might produce more harm than benefit. Furthermore, no study has demonstrated that the addition of MRI to traditional imaging in high-risk women reduces breast cancer-related mortality. In the absence of clear evidence, there is no consistent recommendation from international guidelines on a threshold of breast cancer lifetime risk (BC-LTR) that warrants the recommendation of periodic MRI surveillance [21,22].

In two cohort studies outside the high-risk setting, focused on healthy women who underwent MRI and MX with or without US, MRI had better sensitivity than MX with or without US, particularly in women with dense breasts [11,23]. No interval cancers were observed [11], but the net benefit and additional costs of MRI were not estimated. In the DENSE trial, the supplemental MRI screening in women with extremely dense breasts and negative MX resulted in a significantly lower interval breast cancer rate than MX alone [24].

However, an add-on strategy (i.e., adding tests to MX) to increase the sensitivity of the screening process may decrease specificity and potentially increase overdiagnosis. An alternative strategy, i.e., replacing MX plus US with MRI, could be more risk- and cost effective. This hypothesis has never been explored in a classical head-to-head trial. However, a typical efficacy trial requires the recruitment of tens of thousands of subjects and would require evidence, up to now unavailable, on the acceptability and actual performance of MRI, as a stand-alone screening test among women at intermediate risk of breast cancer are not available. Thus, the MRIB feasibility multicenter, randomized, controlled trial was started in 2013 in Italy, aiming to compare the performance of contrast-enhanced MRI as a stand-alone screening tool versus MX plus US in women at intermediate breast cancer risk and investigate the feasibility of a larger efficacy trial. We here describe the study design and protocol and analyze the distribution of patients’ characteristics and breast cancer risk profiles in the enrolled cohort.

## 2. Study Design and Protocol

This study is in accordance with the ethical standards of the institutional research committees (Ethics Committee of Regione Liguria for the coordinating center and each participating center competent Ethics Committee). All women enrolled in the study received an information sheet and signed written informed consent.

### 2.1. Study Design and Population

Women were randomly assigned to receive annual 2D MX plus US (standard-of-care arm) or MRI (experimental arm) with a 1:1 allocation ratio. Two screening rounds per woman were planned.

Women were deemed eligible for enrollment if aged 40–59 years and if they had a 15–30% BC-LTR and/or extreme BD on the most recent MX. BC-LTR was calculated using the IBIS Breast Cancer Risk Evaluation Tool version 6.0.0 (http://www.ems-trials.org/riskevaluator/, accessed on 30 August 2021).

Exclusion criteria were signs or symptoms of breast cancer, previous breast cancer (invasive or ductal in situ), cancer at any other site, presence of life-threatening diseases, known *BRCA* or *TP53* pathogenic germline mutation, and pregnancy. We also excluded women with general contraindications to MRI or to intravenous administration of gadolinium-based contrast agent; women who underwent hormonal enhancement of ovarian function for medically assisted reproduction in the previous three years; women planning a pregnancy; women undergoing postmenopausal hormone replacement therapy (HRT) who refused to suspend the treatment four weeks before MRI performance.

### 2.2. Enrollment

Women aged 40–59 years who had an MX scheduled during the study period were interviewed to check their eligibility; they were concurrently informed about the study aims and the associated potential risks and benefits. Those who accepted to participate signed informed consent and were randomized. Randomization was centralized via a web-based connection to the coordinating center (http://ctrials.hsanmartino.it/ist/rde/, accessed on 30 August 2021). After eligibility had been checked, the assignment of each woman was disclosed to the center. Randomization was stratified according to center and women’s age at enrollment (<50 or ≥50 years).

### 2.3. Participating Centers and Imaging Readers

According to the EUSOMA recommendations [25], the following facilities had to be available at each participating center: (1) an electronic image storage system for MX, US, and MRI; (2) full-field digital MX systems; (3) breast US scanners; (4) MR units with magnets with field intensity ≥ 1.0 T and gradients ≥ 20 mT/m (details in Table 1). Radiologists involved in the study had to document their experience in breast imaging, i.e., the performance and/or interpretation of at least 500 breast MRI, 10,000 MX, and 5000 US examinations, adequate skill in interventional procedures (under stereotactic or US guidance), and the interpretation of at least 150 breast MRI examinations in the previous year. In addition, participating centers had to guarantee the performance of needle biopsy (core or vacuum-assisted) under stereotactic, US, and MRI guidance; second-look US and reevaluation of MX to identify MRI-detected lesions; availability of preoperative localization under stereotactic, US, and MRI guidance. In total, 10 qualified centers with long experience in breast MRI screening for high-risk women (as in the HIBCRIT study [15]) joined the study, and a team of investigators was established at each center.

### 2.4. Imaging Interpretation

Examinations were interpreted according to the BI-RADS classification system for MX, US, and MRI [26]: category 0 (non-diagnostic), category 1 (negative), category 2 (benign), category 3 (probably benign, i.e., cancer probability < 2%), category 4 (suspicious abnormality—biopsy should be considered) and category 5 (highly suggestive of malignancy—appropriate action should be taken).

Mammographic BD was visually evaluated and categorized according to the following American College of Radiology (ACR) categories: (1) almost entirely fat (*a*), (2) scattered fibroglandular densities (*b*); (3) heterogeneously dense, which may obscure small masses (*c*); (4) extremely dense, which lowers MX sensitivity (*d*) [26]. When the breasts were not of apparently equal BD, the denser one was used to categorize BD.

### 2.5. Diagnostic Workup

In both study arms, women with examinations classified as BI-RADS category 0 repeated imaging tests; those classified as BI-RADS categories 1 or 2 were returned to the assigned arm or to usual screening if the two study rounds had been completed. Women with examinations classified as BI-RADS category 4 or 5 were immediately invited to undergo further diagnostic and/or interventional procedures, as appropriate.

Women who had MX and/or US examinations classified as BI-RADS category 3 were invited to repeat MX and/or US within 6 months (early recall) according to the characteristics of the detected lesion(s). If the early recall exams were classified as BI-RADS category 1 or 2, women returned to the assigned group or to screening; if they were classified as BI-RADS category 3 to 5, the women were invited to undergo further diagnostic and interventional procedures.

Women who had MRI classified as BI-RADS category 3 were referred to second-look US and/or reevaluation of MX according to the characteristics of the observed lesion(s). Those who had the second-look/reevaluation test(s) classified as BI-RADS category 1 or 2 were returned to the assigned study arm or to screening. Women with test(s) classified as BI-RADS category 3 were invited to repeat MX and/or US after 3 months; short-term follow-up with MRI was not considered. If the result of the 3-month examination(s) remained BIRADS category 3 or worsened to category BI-RADS 4, or 5, the women were invited to undergo interventional procedures.

**Figure 1:**
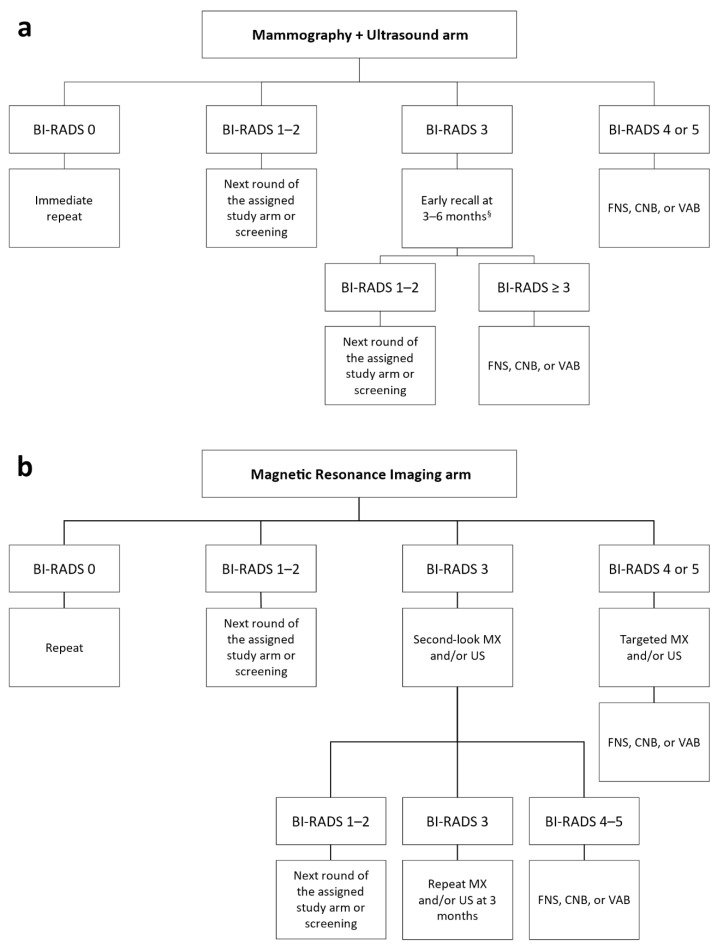
(**a**) Diagnostic workup for women assigned to the standard-of-care arm with suspicious mammography and/or ultrasound; (**b**) diagnostic workup for women assigned to the experimental arm with suspicious breast magnetic resonance imaging. ^§^ According to the characteristics of the detected lesion(s). BI-RADS: Breast Imaging Reporting and Data System; FNS: fine-needle sampling; CNB: core-needle biopsy; VAB: vacuum-assisted biopsy; MX: mammography; US: ultrasound.

Diagnostic and interventional procedures performed in the workup of detected abnormalities (either after screening examination or after short-term follow-up) included fine-needle sampling, core-needle biopsy with at least 14 g bore devices with or without coaxial systems, and vacuum-assisted biopsy with at least 11 g bore devices. The diagnostic workup in the two study arms is reported in Figure 1.

### 2.6. Data Collection

The following data were collected upon enrollment of each woman: reproductive history, use of birth control pill, HRT, height, weight, alcohol consumption, tobacco smoking history, first-degree family history of breast cancer and of ovarian cancer (OC), ACR density class at the most recent MX, and BC-LTR.

The coordinating center was responsible for data storage, monitoring, and quality controls of the study, as well as for the assessment of main study endpoints. The Clinical Trials Center of the coordinating center developed the electronic case report forms to record: imaging data examinations, pathology data for lesions biopsied and/or removed, details of surgical procedures, stage of detected breast cancer, eventual adverse events. A password-protected database was designed and managed, each researcher receiving a personal login/password. The Clinical Trials Center was also in charge of monitoring data collection and auditing the filled-in case report forms. Participating centers provided de-identified data according to current regulations. At the Clinical Trials Center, the enrolled women were identified with a unique study number assigned at randomization.

### 2.7. Study Endpoints

The primary endpoint of this study was the rate of invasive and ductal in situ breast cancer detected in the two study arms. All breast cancers diagnosed as a consequence of an abnormality identified at the screening tests were considered screen detected.

Secondary endpoints included (1) the distribution of clinical and pathological stages of invasive breast cancers; (2) the histological characteristics of breast cancers; (3) the interval cancer rate, both between the two examinations and within one year from the second examination: any breast cancer (invasive or ductal in situ) diagnosed after a negative examination but before the following examination, scheduled approximately 1 year later, would be considered as an interval cancer; (4) the adherence to the assigned arm and any reason for consent withdrawal from the assigned program; (5) the distribution of the breast cancer risk profiles; (6) the number of breast cancers (invasive and in situ) detected in excess in the experimental arm compared to the conventional arm (overdiagnosis) after 4 years of follow up. The breast cancer risk profile was built combining the LTR score (<15% or ≥15%) and the BD at the most recent mammography before enrollment (ACR class *a* to *c*, or ACR class *d*), so that four risk categories (risk profile) were created: (1) LTR ≥ 15% and BD = *a* to *c*; (2) LTR < 15% and BD = *d*; (3) LTR ≥ 15% and BD = *d*; (4) LTR ≥ 15% and unknown BD.

### 2.8. Sample Size Estimation

The study was designed as a feasibility study, preliminary to the conduction of a large-size efficacy trial. The size of an efficacy trial with breast cancer mortality (or incidence of metastatic breast cancer) as the primary endpoint should allow the observation of at least 380 events (breast cancer deaths, or incidence of metastatic breast cancer) in order to detect—with an 80% power—a 25% relative reduction in breast cancer mortality, which is considered the minimal effect of MRI screening that is worth detecting. Assuming a 60% survival at 10 years [27], and an average cumulative 10-year breast cancer risk of 5% (the lowest risk in this cohort should be about 3%) [22], we can estimate that at least 20,000 women followed for 10 years (with a further follow-up of breast cancers until 380 events have been observed) would be necessary for such an efficacy trial. However, accrual for large-size prevention trials is very difficult, as they target asymptomatic healthy individuals facing a variable but generally low risk. Thus, this feasibility trial aimed to estimate the sensitivity and specificity of MRI screening alone but also to provide information on the distribution of the risk profiles among enrolled women, as well as to estimate the sample size needed by an efficacy trial. Therefore, a planned enrollment of 2000 women (10% of the size of the efficacy trial) was proposed. Furthermore, organizational problems and quality control issues could be adequately addressed in a study of this size.

It can be expected that in this feasibility trial, over a 5-year screening period, about 40–60 cases of invasive breast cancer will be observed: one-third in the control arm; two-thirds in the MRI arm. These figures are close to those of previous uncontrolled MRI studies (e.g., the HIBCRIT study [15]) and would enable us to confirm the twofold increase in sensitivity associated with MRI. These figures were also considered sufficient to provide preliminary information on the MRI-associated lead time and on the stage distribution of incident breast cancers. Conversely, the number of advanced (metastatic or locally advanced) breast cancers and the number of breast-cancer-related deaths should be too small to allow any meaningful interpretation. Due to lead time, no effects of MRI on efficacy endpoints are expected to be noted during the first years. Any estimation of the proportion of interval cancers in each arm proved to be difficult, as this proportion is dependent on the age distribution of enrolled women.

Two and half years since the start of the study, when 1254 women had been randomized, enrollment stopped due to the end of the grant, and screening and diagnostic imaging was completed. A clinical follow-up of all randomized women is planned for at least 5 years.

## 3. Characteristics of Enrolled Patients

From July 2013 to November 2015, a total of 1254 women (mean age 47.2 ± 4.6 years) were enrolled: 624 were assigned to MX plus US (standard of care arm) and 630 to MRI (experimental arm). Table 2 reports the number of women recruited in each center, and their characteristics are detailed in Table 3.

Most women were below the age of 50 (72.2%) and premenopausal (44.6%) or perimenopausal (23.7%). More than one in four (27.6%) did not have children; 52.1% used oral contraceptives (currently or in the past); 15% of postmenopausal women had used HRT. Most women (86.3%) had a body mass index <25. Only 4.6% of enrolled women (58/1254) had their first breast examination in this trial, and most of them (44/58) were in their forties. Among women with a previous MX, an extremely dense breast at the most recent MX was recorded for 82.7% (984/1196). Breast and/or ovarian cancer in mothers and/or sisters was reported by 36.5% of enrolled women (458/1254) and 67 of them had a BC-LTR < 15%. A BC-LTR ranging from 15% to 30% was calculated for 41% (514/1254) of all enrolled patients, and 47.5% of them (244/514) also had a previous MX classified as extremely dense. Overall, 59% of enrolled women (740/1254) had, as a sole inclusion criterion, an extremely dense breast at MX (LTR < 15% and BD = *d*).

The distribution of major determinants of breast cancer risk profiles of women enrolled in the study varied across centers: the rate of women recruited on the basis of an MX classified as extremely dense ranged from 0% to 98.9% (Table 4).

The frequency of women reporting one or more first-degree relatives affected by breast and/or ovarian cancer ranged from 26.6% to 91.7%.

## 4. Discussion

In Italy, women aged 50–69 years are offered biennial screening MX independently of their BD and BC-LTR. Currently, an increasing number of women opt for regular surveillance imaging from age 40 onwards, particularly when they perceive being at increased breast cancer risk [28]. Outside organized screening, women with high BD are usually offered yearly MX plus US, even though supplemental US increases false-positive findings and data concerning the benefit of US supplemental screening in terms of reduced interval cancer rates are not consistent [20,29,30,31]. In addition, it is not known whether the increased breast cancer detection by US translates into reduced mortality.

Our study focused on intermediate-risk women aged 40–59 years, only partially targeted by organized screening programs. To our knowledge, this is the first trial testing breast MRI as a stand-alone screening tool, compared to MX plus US. The rationale stemmed from observational studies on high-risk populations, that showed a twofold increase in MRI sensitivity, compared to MX/US, but without a significant increase in sensitivity from the addition of MX/US to MRI [14,15,32,33]. The effect of an increased detection on breast cancer-related mortality and the entity of any MRI-associated overdiagnosis can only be addressed with RCTs, which are, however, difficult to conduct in high-risk settings due to ethical and psychological reasons. Therefore, two options are available: (1) to rely on ongoing and future uncontrolled studies, whose validity is undermined by biases affecting the comparison with external controls; (2) to conduct RCTs in women to whom MRI screening is not currently offered. The latter approach has two limitations: (1) due to the lower breast cancer risk, sample sizes will have to be much larger; (2) the results will not be directly applicable to women to whom MRI screening is currently offered, due to different risk profiles and possibly also different breast cancer biology. However, these two options are not mutually exclusive, and both can give information on benefits, risks, harms, and costs associated with screening MRI. This would be especially relevant considering the two major issues encumbering breast MRI screening—namely, patient compliance and high costs related to instrumental, technical (contrast agent, acquisition time, post-processing), and interpretation aspects [34,35]. Notably, regarding breast MRI screening uptake, Berg et al. [36] reported that over 40% of women at high breast cancer risk refused to undergo additional MRI screening; a similar result was observed in the DENSE trial [24]; as for the cost–benefit analysis, new studies with larger temporal horizons have recently highlighted a better outlook [37,38], which could be reinforced by the introduction of abbreviated protocols reducing acquisition and interpretation times, now known to match the accuracy of full protocols [39,40,41].

Our population comprised healthy women with a 2–3-fold increase in breast cancer risk, compared to a low-risk woman of the same age. We assessed BC-LTR with the IBIS Breast Cancer Risk Evaluation Tool version 6.0.0, which incorporates the most comprehensive set of personal risk factors and an extensive family history of breast and ovarian cancer. Since the IBIS Breast Cancer Risk Evaluation Tool does not integrate BD, we set extreme BD as an eligibility criterion. Over 70% of enrolled women were in their 40s, the age range in which screening recommendations are not consistent across Europe [42]. Due to recruitment at radiology units, 95% of enrolled women had a pre-trial MX, but the distribution of risk profiles varied widely across centers (from 0% to 99% of women having extremely dense breasts), showing different ultimate sources of recruitment (e.g., self-referral for breast examination or collaboration with familial cancer clinics for the surveillance of women at increased breast cancer risk who do not carry pathogenic germline variants). In our study, around one in four women with extreme BD had also a BC-LTR ≥ 15%: in this subgroup, a BC-LTR recalculation using the 2017 IBIS Breast Cancer Risk Evaluation Tool version 8b that includes BD in the model might take the risk over the 30% threshold in a high number of cases.

Our study has some limitations: it was designed as a preliminary, feasibility study with the goal of recruiting 2000 women in 2.5 years, but in the designed period, we recruited only 1254 women. Each center was required to enroll 200 women, but only 3 out of 10 centers reached the target. We observed that the study budget did not adequately incorporate staff needed to support such a study; for instance, significant workflow demands fell on the radiologists, as one of the required assessments for eligibility was the evaluation of previous MX with subsequent BC-LTR calculation. Time constraints and concurrent competing trials were causes of the failure to reach the expected recruitment. As in other trials, inadequate funding and complexity of the study design were the reasons that contributed to unsuccessful trial recruitment [43]. Moreover, as is common when dealing with first-round MRI, we faced a high rate of BI-RADS 3 designations. Especially in the case of “clearly” intermediate-risk population (greatly lowering the pre-test cancer probability in comparison with *BRCA* or *TP53* mutation carriers) and in the absence of any correlate at reassessed MX and targeted US, we hypothesized that the residual cancer rate could be sufficiently low to postpone the MRI to the year after. This approach has been already investigated by Elshof et al. [44] for additional MRI-detected lesions outside the primary tumor region in the preoperative setting, where the pre-test cancer probability should be higher than in any screening setting. Additional lesions outside the primary tumor region without any imaging correlate at targeted US were found in 81 out of 690 patients. None of them resulted in malignant disease at follow-up after breast-conserving therapy (mean follow-up time: 57.1 months). To minimize the risk of a diagnostic delay, we also planned a 3-month follow-up with MX and US. Furthermore, we highlight that our intermediate screening setting implied an expected low cancer detection rate: researchers had to reckon also with this expectation, trying to minimize unnecessary biopsies and overdiagnosis [13,45,46,47]. Finally, researchers had also to be aware of the peculiar spectrum of potential false-positive and false-negative findings, for example, those engendered by motion artifacts [48] and by the influence of background parenchymal enhancement [49,50], which are commonly associated with MRI. As mentioned above, general countermeasures to curtail them, such as the repetition of MX or the use of targeted US, were implemented in this study and will be the object of specific analysis. In addition, to this purpose, future studies on the application of MRI in similar settings could benefit from the use of technological advances (such as fusion imaging [51]) or combined prediction models [52].

## 5. Conclusions

The rationale of the MRIB trial stems from the general need to acquire a more accurate understanding of the costs and benefits associated with the extension of MRI screening outside the high-risk setting. While other large-scale studies such as the DENSE trial [24] explored the use of MRI in the screening setting as a supplemental examination, the MRIB trial compares MRI alone to MX+US, in an effort to balance diagnostic performance, risk-effectiveness, and cost-effectiveness. Pioneering the stand-alone use of MRI in the intermediate-risk setting, this trial has a feasibility design that, despite the main limitations of its preliminary nature and of the inability to reach the expected recruitment, will allow us to provide useful information on the acceptability of the two screening models and on their diagnostic performance in terms of second-look examinations, short-term reevaluation, invasive procedures, and diagnostic yield. Furthermore, data from the planned 5-year follow-up should allow the estimation of the magnitude of overdiagnosis, if any, associated with MRI. In a broader timeframe, the MRIB trial will also contribute data for the informed performance of cost–benefit analyses and, potentially, for the design and planning of a formal head-to-head efficacy trial of MRI screening in women at intermediate breast cancer risk.

## Figures and Tables

**Table 1:** General requirements for radiology departments to participate in the MRIB study.

**1**	Availability of an electronic image storage system for MX, US, and MRI
**2**	Full-field digital MX systems with high-resolution electronic display systems available to both the technologist at the time of the examination and to the interpreting physician. Mandatory availability on the display settings of the dedicated workstation of relevant information about the digital images and the examined patient.
**3**	Breast US scanners equipped with a multi-frequency linear array transducer operating at a center frequency higher than 10 MHz.
**4**	MR units with magnets with intensity field ≥ 1.0 T and gradients ≥ 20 mT/m, equipped with bilateral dedicated coils (preferably multichannel) and an automated power injector system with double syringe for both contrast agent and normal saline solution. The MRI protocol must include a high-contrast bilateral morphologic sequence and a bilateral dynamic two-dimensional or three-dimensional study with spatial in-plane resolution ≤ 1.5 mm^2^ (preferably ≤ 1 mm^2^) and temporal resolution ≤ 120 s.

**Table 2:** Distribution of the 1254 randomized women according to study arm among recruitment centers.

Center	Mammography Plus Ultrasound *n* (%)	Magnetic Resonance Imaging *n* (%)
IRCCS Ospedale Policlinico San Martino, Genova	139 (22.3%)	139 (22.1%)
Ospedale Universitario Sant’Anna, Cona—Università degli Studi di Ferrara, Ferrara	100 (16.0%)	101 (16.0%)
Azienda Ospedaliera Universitaria Integrata, Verona	100 (16.0%)	100 (15.9%)
IRCCS Policlinico San Donato, San Donato Milanese	53 (8.5%)	54 (8.6%)
IRCCS Ospedale San Raffaele, Milano	50 (8.0%)	50 (7.9%)
Dipartimento di Scienze Radiologiche, Oncologiche, Patologiche—Università La Sapienza, Roma	45 (7.2%)	45 (7.1%)
Azienda Ospedaliera Universitaria “Santa Maria della Misericordia”, Udine	39 (6.2%)	39 (6.2%)
Fondazione IRCCS Istituto Nazionale dei Tumori, Milano	34 (5.4%)	38 (6.0%)
Fondazione Policlinico Universitario Agostino Gemelli IRCCS—Università Cattolica del Sacro Cuore, Roma	34 (5.4%)	34 (5.4%)
Istituto Nazionale Tumori IRCCS Fondazione G. Pascale, Napoli	30 (4.8%)	30 (4.8%)
Total	624 (100.0%)	630 (100.0%)

**Table 3:** Characteristics and risk profiles of the 1254 randomized women according to study arm.

	Mammography Plus Ultrasound *n* (%)	Magnetic Resonance Imaging *n* (%)
Total	624 (100.0%)	630 (100.0%)
**Age classes**		
40–44	214 (34.3%)	206 (32.7%)
45–49	236 (37.8%)	249 (39.5%)
50–54	130 (20.8%)	121 (19.2%)
55–59	44 (7.1%)	54 (8.6%)
**Age at menarche (years)**		
≤11	118 (18.9%)	141 (22.4%)
12–13	336 (53.8%)	311 (49.4%)
≥14	165 (26.4%)	171 (27.1%)
Unknown	5 (0.8%)	7 (1.1%)
**Number of full-term pregnancies**		
0	170 (27.2%)	176 (27.9%)
1	208 (45.8%)	176 (38.8%)
2	211 (46.5%)	231 (50.9%)
≥3	35 (7.7%)	47 (10.4%)
**Age at first birth (years)**		
<20	4 (0.9%)	9 (2.0%)
20–24	65 (14.3%)	62 (13.7%)
25–29	124 (27.3%)	140 (30.9%)
30–34	141 (31.1%)	128 (28.3%)
≥35	101 (22.2%)	98 (21.6%)
Unknown	19 (4.2%)	16 (3.5%)
**Menopausal status**		
Premenopausal	282 (45.2%)	277 (44.0%)
Perimenopausal	150 (24.0%)	147 (23.3%)
Postmenopausal	117 (18.8%)	130 (20.6%)
Unknown	75 (12.0%)	76 (12.1%)
**Contraceptive pill use**		
Never	303 (48.6%)	298 (47.3%)
Current	59 (9.5%)	60 (9.5%)
Discontinued < 5 years	39 (6.2%)	46 (7.3%)
Discontinued ≥ 5 years	223 (35.7%)	226 (35.9%)
**HRT use in post-menopause**		
Never	104 (88.9%)	105 (80.8%)
Past	9 (7.7%)	21 (16.1%)
Current	4 (3.4%)	4 (3.1%)
**Body mass index (kg/m^2^)**		
<25	523 (83.8%)	525 (83.3%)
25–29	68 (10.9%)	63 (10.0%)
≥30	12 (1.9%)	17 (2.7%)
Unknown	21 (3.4%)	25 (4.0%)
**Cigarette smoking**		
Never	420 (67.3%)	425 (67.5%)
Past	107 (17.1%)	97 (15.4%)
Current	97 (15.5%)	108 (7.1%)
**Alcohol consumption**		
Never	446 (71.5%)	441 (70.0%)
Past	39 (6.2%)	41 (6.5%)
Current	139 (22.3%)	148 (23.5%)
**First-degree family history of breast cancer and ovarian cancer**		
None	383 (61.4%)	413 (65.6%)
Breast cancer	218 (34.9%)	197 (31.3%)
Ovarian cancer	17 (2.7%)	16 (2.5%)
Breast cancer and ovarian cancer	6 (1.0%)	4 (0.6%)
**Breast density at pre-trial MX**		
*a*	6 (1.0%)	9 (1.4%)
*b*	47 (7.5%)	53 (8.4%)
*c*	56 (9.0%)	41 (6.5%)
*d*	486 (77.9%)	498 (79.0%)
Unknown	29 (4.6%)	29 (4.6%)
**LTR of breast cancer**		
<15%	358 (57.4%)	382 (60.6%)
15–30%	266 (42.6%)	248 (39.4%)
**Risk profile**		
LTR ≥ 15%, breast density (*a*–*c*)	109 (17.5%)	103 (16.3%)
LTR < 15%, breast density (*d*)	358 (57.4%)	382 (60.6%)
LTR ≥ 15%, breast density (*d*)	128 (20.5%)	116 (18.4%)
LTR ≥ 15%, unknown breast density	29 (4.6%)	29 (4.6%)

MX, mammography; HRT, hormone replacement therapy; LTR, lifetime risk. Breast density at the pre-trial MX: (*a*) almost entirely fat, (*b*) scattered fibroglandular densities, (*c*) heterogeneously dense, and (*d*) extremely dense.

**Table 4:** Distribution of the risk profiles of women enrolled in the 10 participating centers.

	**Breast Cancer LTR < 15%**	**Breast Cancer LTR 15–30%**		
Centers	Breast Density (*d*) *n* (%)	Breast Density Not Assessed *n* (%)	Breast Density (*a*–*c*) *n* (%)	Breast Density (*d*) *n* (%)
IRCCS Ospedale Policlinico San Martino, Genova	230 (82.7%)	2 (0.7%)	1 (0.4%)	45 (16.2%)
Ospedale Universitario Sant’Anna, Cona—Università degli Studi di Ferrara, Ferrara	115 (57.2%)	0 (0.0%)	53 (26.4%)	33 (16.4%)
Azienda Ospedaliera Universitaria Integrata, Verona	132 (66.0%)	0 (0.0%)	3 (1.5%)	65 (32.5%)
IRCCS Policlinico San Donato, San Donato Milanese	72 (67.3%)	0 (0.0%)	26 (24.3%)	9 (8.4%)
IRCCS Ospedale San Raffaele, Milano	21 (21.0%)	39 (39.0%)	20 (20.0%)	20 (20.0%)
Dipartimento di Scienze Radiologiche, Oncologiche, Patologiche—Università La Sapienza, Roma	58 (64.4%)	15 (16.7%)	4 (4.4%)	13 (14.4%)
Azienda Ospedaliera Universitaria “Santa Maria della Misericordia”, Udine	33 (42.3%)	2 (2.6%)	23 (29.5%)	20 (25.6%)
Fondazione IRCCS Istituto Nazionale dei Tumori, Milano	30 (41.7%)	0 (0.0%)	22 (30.6%)	20 (27.8%)
Fondazione Policlinico Universitario Agostino Gemelli IRCCS—Università Cattolica del Sacro Cuore, Roma	49 (72.1%)	0 (0.0%)	0 (0.0%)	19 (27.9%)
Istituto Nazionale Tumori IRCCS Fondazione G. Pascale, Napoli	0 (0.0%)	0 (0.0%)	60 (100.0%)	0 (0.0%)
Total	740 (59.0%)	58 (4.6%)	212 (16.9%)	244 (19.5%)

LTR, lifetime risk. Breast density: (*a*) almost entirely fat, (*b*) scattered fibroglandular densities, (*c*) heterogeneously dense, and (*d*) extremely dense.

## Data Availability

This article details the study design, protocol, and characteristics of patients enrolled in the MRIB multicenter, randomized, controlled trial. All remaining study data will be the object of separate publications.
